# Correction: Gupta et al. Androgen Receptor Activation Induces Senescence in Thyroid Cancer Cells. *Cancers* 2023, *15*, 2198

**DOI:** 10.3390/cancers16213636

**Published:** 2024-10-29

**Authors:** Anvita Gupta, Michelle Carnazza, Melanie Jones, Zbigniew Darzynkiewicz, Dorota Halicka, Timmy O’Connell, Hong Zhao, Sina Dadafarin, Edward Shin, Monica D. Schwarcz, Augustine Moscatello, Raj K. Tiwari, Jan Geliebter

**Affiliations:** 1 Department of Pathology, Microbiology, and Immunology, New York Medical College, Valhalla, NY 10595, USA; 2 Department of Medicine, New York Medical College, Valhalla, NY 10595, USA; 3 Department of Otolaryngology-Head and Neck Surgery, University of Washington, Seattle, WA 98195, USA; 4 Department of Otolaryngology, New York Eye and Ear Infirmary of Mount Sinai, New York, NY 10003, USA; 5 Department of Medicine, New York University Grossman School of Medicine, New York, NY 10016, USA; 6 Department of Otolaryngology, New York Medical College, Valhalla, NY 10595, USA

## Error in Figure

In the original publication [1], there was a mistake in Figure 11 as published. Figure 11A had two overlapping images in the DHT samples. The corrected Figure 11 appears below. The authors state that the scientific conclusions are unaffected. This correction was approved by the Academic Editor. The original publication has also been updated.


